# Breast cancer cells and adipocytes in hypoxia: metabolism regulation

**DOI:** 10.1007/s12672-024-00865-w

**Published:** 2024-01-18

**Authors:** Xin Yu, Tianqi Zhang, Xiaozhi Cheng, Li Ma

**Affiliations:** The Fourth Hospital of Hebei Medical University, Shijiazhuang, Hebei China

**Keywords:** Adipocytes, Breast cancer, HIF-1α, Metabolism

## Abstract

Adipocytes play a significant role in breast cancer due to the unique histological structure of the breast. These have not only been detected adjacent to breast cancer cells but they have also been implicated in cancer development. Adipocytes in obese individuals and tumor microenvironment (TME) have a common feature, that is, hypoxia. The increased expression of hypoxia-inducible factor (HIF)-1α is known to alter the metabolism and functions of adipocytes. In this study, we described the mechanism linking the hypoxia-sensing pathway manifested by HIF to adipocytes and breast cancer and discussed the mechanism underlying the role of hypoxic adipocytes in breast cancer development from the perspective of metabolic remodeling. The processes and pathways in hypoxic adipocytes could be a promising target in breast cancer therapy.

## Introduction

Breast cancer is the most commonly diagnosed cancer in women and has also surpassed lung cancer. It is one of the five most deadly cancers worldwide, accounting for an estimated 1 in 4 cancer cases and 1/6 of cancer deaths [1]. Although the intrinsic characteristics of cancer cells contribute to the development of breast cancer, the tumor microenvironment (TME) and individual differences play a crucial role as well. Adipose tissue is a highly active endocrine organ that releases adipokines to regulate several physiological processes such as energy production, neuroendocrine functions, immune regulation, and reproduction [2]. In addition, it contributes to the growth and development of the breasts [3] and is involved in the changes of breasts at different ages [4]. Studies have reported an association between adipocytes and breast cancer that leads to increased tumor invasion, migration, and drug resistance [5]. Hypoxia-inducible factor (HIF)-1α is a key mediator of hypoxia, whose activation causes adipocyte dysfunction and metabolic abnormalities [6–8], consequently affecting cancer progression. In this review, we described the effects of adipose tissue on breast development and explored the effects of adipocytes on breast cancer under hypoxic conditions from the perspective of metabolic reprogramming.

## Adipose tissue in the breast

As an endocrine organ, adipose tissue releases different adipokines such as leptin and adiponectin to regulate several physiological processes [9], such as energy production, neuroendocrine functions, immune regulation, and reproduction. In the breast, the percentage of adipose tissue volume to total breast volume varies from 7 to 56%, and adipose tissue weight accounts for 3.7–37% [10]. Adipokines released by the adipose tissue in the breasts induce the differentiation of the mammary epithelium and participate in the development of the breasts. Furthermore, adipocytes in the breasts undergo differentiation during pregnancy and lactation. During pregnancy, white adipocytes in the breast differentiate into milk-producing glands with abundant lipids through the integrin-secreted phosphoprotein 1 (SPP1) signaling pathway and are termed pink adipose tissue [11]. During pregnancy, the activated peroxisome proliferator-activated receptor γ (PPARγ) promotes the reverse conversion of pink adipose into white adipose [12]. Thus, mammary adipose tissue not only contributes to the development of the whole mammary gland development but also participates in the whole process of mammary tissue during pregnancy, lactation, and degeneration.

## Adipose tissue and breast cancer

The interaction between adipocytes and breast cancer is a significant factor driving breast cancer invasion and metastasis; this interaction occurs during all stages of breast cancer progression [13]. Pathological conditions are characterized by adipose tissue hypoxia that can promote the pro-tumor environment in the breast.

The hypoxic state of adipose tissue in obese individuals is associated with inadequate perfusion due to reduced capillary density in the tissue, reduced oxygen diffusion owing to increased cell size, and inadequate oxygen supply because of elevated adipocyte oxygen consumption. Adipocyte hypertrophy causes capillary obstruction and reduces blood flow, causing inflammation, which further reduces blood flow by damaging the capillary endothelium and making it dysfunctional [14]. In addition, the excessive diameter of hypertrophic adipocytes does not allow oxygen to diffuse properly, thereby reducing the availability of oxygen to adipocytes. Saturated fatty acids could stimulate adenine nucleotide translocase, which increases non-coupled mitochondrial respiration, thereby increasing the consumption of adipocyte oxygen [15]. Thus, adipocytes do not have access to adequate blood flow and oxygen, and hypoxia and ischemia can cause a further elevation in oxidative stress in adipocytes, producing a range of carcinogenic effects.

## Hypoxia-inducible factor-1α expression in breast cancer

HIF-1α is a transcription factor activated by obese adipose tissue under hypoxic conditions. Under normoxic conditions, prolyl hydroxylase-2 (PHD-2) hydroxylates proline residues on the N-TAD of HIF-1α, which triggers the interaction of HIF-1α with von Hippel-Lindau tumor suppressor protein (VHL). Subsequently, the binding of ubiquitination ligase leads to the ubiquitination and degradation of HIF-1α under the action of 26s protease [16].

During hypoxia, HIF hydroxylation and ubiquitination are inhibited, causing its accumulation in cells and inducing the transcription of multiple hypoxia-responsive genes, which, consequently, regulate the progression of breast cancer.

HIF-1α affects glucose metabolism in tumor cells by inducing the genes encoding glucose transporter 1 (GLUT1) and glycolytic enzymes [17]. Among the factors affecting tumor angiogenesis, the most critical one is vascular endothelial growth factor (VEGF), and HIF-1α affects tumor cell glucose metabolism by regulating VEGF, hepatocyte growth factor (HGF), and vascular cell adhesion molecule 1 (VCAM1) [18], whereas HIF-1α increases the expression of matrix metalloproteinase-2 (MMP-2) and MMP-9. The expression of MMP-9 disrupts the extracellular matrix and transcribes integrins to promote the targeted movement of breast tumor cells [19], eventually inducing tumor invasion and metastasis. Hypoxia in the TME significantly contributes to tumor immune escape; HIF-1α causes tumor immune escape by promoting macrophage polarization [20], inhibiting the recruitment of regulatory T cells [21], and suppressing the anti-tumor activity and immune cell infiltration of CD8^+^ T cells [22]. Recent studies have shown that the NF-κB, RAS-RAF-MEK-ERK, PI3K/Akt/mTOR, and JAK-STAT signaling pathways regulate the expression of HIF-1α, which in turn drives the biological processes of breast cancer cell proliferation, angiogenesis, and BCSCs enrichment [23–26].

HIF-1α is regulated by a complex network and drives the development of breast cancer through glycolysis, metastasis, angiogenesis, breast cancer stem cells enrichment and activation, and immune escape, indicating that targeting HIF-1α is of great significance for the treatment of breast cancer.

## Adipose tissue affects breast cancer by promoting multiple metabolic pathways

### Glycolysis

The majority of tumor cells obtain ATP under aerobic conditions primarily through the glycolytic pathway—known as the Warburg effect. HIF-1α not only induces the expression of GLUT to uptake extracellular glucose [17] but also increases glycogen synthesis and catabolism, allowing cancer cells to produce sufficient ATP and metabolic intermediates for the synthesis of nucleotides, amino acids, and fatty acids that can provide energy and biosynthetic substrates to neighboring cells [27].

A “reverse Warburg effect” exists between breast cancer cells and adipocytes. During this effect, byproducts of adipocyte glycolysis, such as lactate, are excreted from adipocytes by monocarboxylate transporter 4 (MCT4), which is taken up by tumor cells in response to MCT1 and used for mitochondrial oxidative phosphorylation in breast cancer cells [28]. Furthermore, differential expression of MCT between breast cancer cells and adipocytes enhances the invasiveness of breast cancer. The highly proliferative estrogen receptor-negative breast cancer subtype expresses high levels of MCT1 and is related to poor outcomes in patients [29].

Hypoxia is the primary driver of glycolysis and lactate production. Hypoxia facilitates lactate production in adipocytes, and approximately 50 to 70% of glucose is converted to lactate in adipose tissue. Large amounts of lactate, CO_2_, and other metabolites accumulate in the TME to create an acidic environment, which suppresses immune cell functions and allows tumor cells to escape the immune system.

**Fig. 1:**
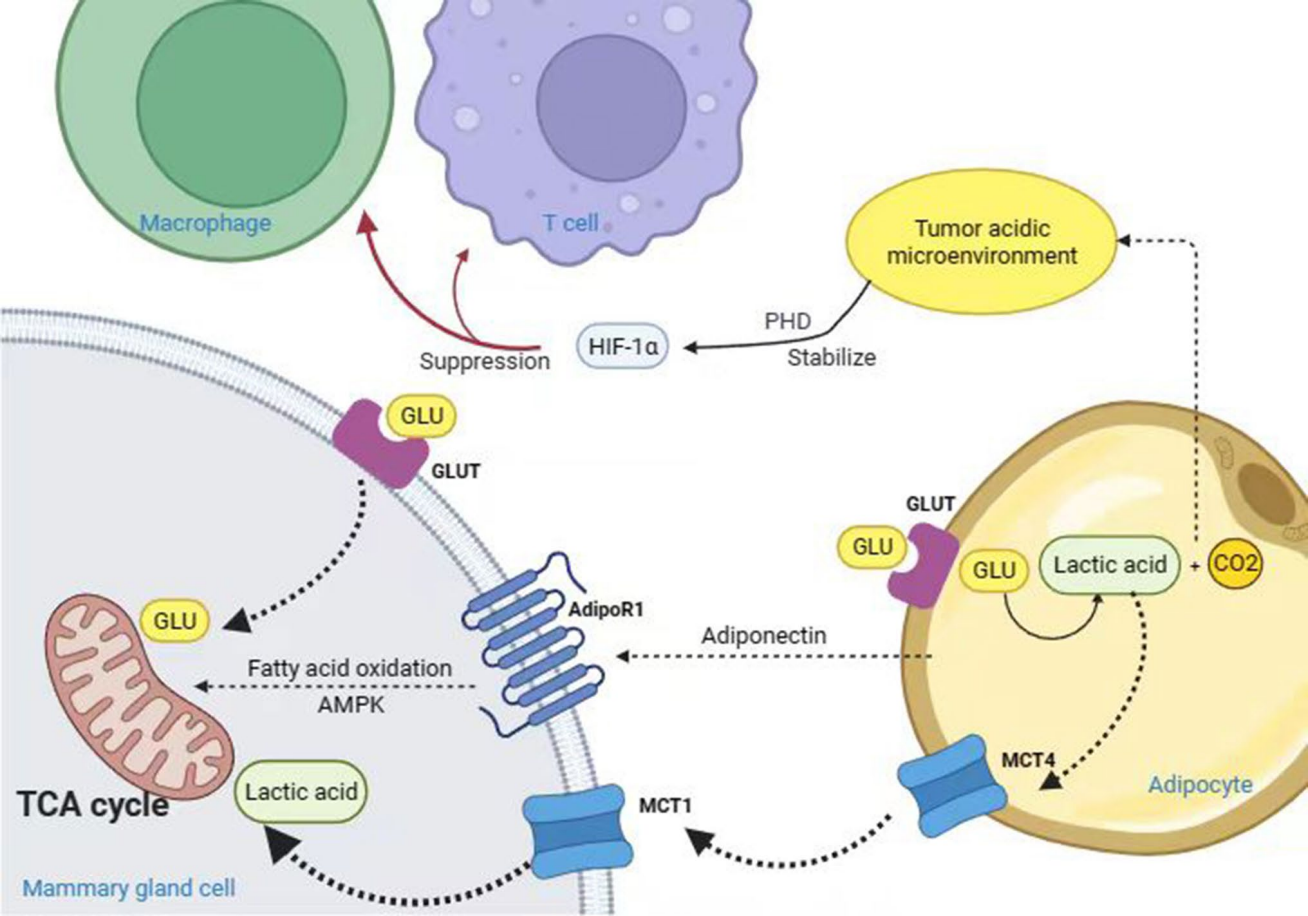
Adipose tissue affects breast cancer by promoting multiple metabolic pathways

Studies have demonstrated that adipocyte-derived lactate contributes to developing an adipose pro-inflammatory microenvironment. This could be because first, an increase in the levels of intracellular lactate initiates apoptosis of adipocytes, thereby initiating an inflammatory response [30]. Second, lactate binds to the catalytic structural domain containing PHD2, thereby stabilizing HIF-1α and reducing its degradation [31]. Third, lactate induces M1 macrophages to polarize to the M2 type, causing immunosuppression and tissue remodeling in the tumor zone (as shown in Fig. 1) [32].

In summary, in breast cancer, HIF-1α mainly promotes glycolysis by inducing genes encoding glucose transporters such as GLUT1 and glycolytic enzymes.

### Lipid metabolism

Under hypoxia, both breast and ovarian cancer cells enhance the rate of adipocyte lipolysis [33]. Cohen’s team found through RNA-seq that the para-cancerous adipose metabolism pathway changed significantly, high levels of creatine are released from adipocytes and taken up by adjacent cancer cells, mainly through the Slc6a8 and Smad2/3 pathways, thereby promoting the progression of breast cancer [34].

A study demonstrated that following co-culturing of breast cancer cells with adipocytes, adipocyte-derived free fatty acids were transferred into breast cancer cells. Through the AMPK pathway, elevating the levels of carnitine palmitoyl transferase 1 (CPT1A) and electron transfer proteins [35], consequently driving fatty acid metabolism to maintain a high level of ATP and enhance the ability of breast cancer tumor cells to grow, survive, and multiply.

Both adipocyte-released lipids and lipids entering the cancer cells are reduced in HIF-deficient adipocytes, such that the lipid availability of such adipocytes is low. Therefore, the release of fatty acids by adipocytes and subsequently reuptake by cancer cells depends on the hypoxic HIF-1α pathway.

The above indicated that fatty acid secreted by obese fat plays an important role in breast cancer proliferation. Inhibiting the production of FASN may be one of the directions for the treatment of breast cancer in the future.

## Combined application of targeted HIF-1α for diet control, drugs, chemotherapy and immunotherapy

During cancer treatment, dysregulation of cell metabolism in tumor areas is associated with drug resistance. HIF-associated metabolic pathways participate in the development of adipocyte-associated cancers.

Several therapeutic targets can be proposed for HIF-dependent metabolic reprogramming, such as reducing adipose tissue, altering adipocyte metabolic mode, and targeting HIF to prevent or treat breast cancer. Multiple therapeutic approaches are available to target HIF-1α, such as inhibiting the regulation of upstream target genes of HIF-1α, modulating protein modifications to promote HIF-1α protein hydroxylation and degradation, inhibiting HIF-1α protein synthesis, and inhibiting the regulation of downstream target genes of HIF-1α.

Diet control is the simplest method to lose fat. In addition, reducing caloric intake regulates the levels of relevant hormones in the body to protect postmenopausal women with breast cancer. In the WHI DM trial published in 2020, a dietary intervention in 48,835 postmenopausal women without previous breast cancer, a significant reduction in breast cancer deaths persisted after a long-term cumulative 19.6 years of follow-up (359 [0.12%] v 652 [0.14%] deaths) [36]. Low-fat diets prevent and treat breast cancer by reducing the production and secretion of adipokines and insulin, enhancing immunity, and diminishing drug resistance [37]. Certain substances extracted from broccoli, soybean, and ginger can hinder tumor progression by promoting the self-renewal of mesenchymal stem cells, preventing the differentiation of preadipocytes into mature adipocytes, and inhibiting the transcription levels of HIF.

Cardamonin is a flavonoid that increases the sensitivity of tumors to chemotherapeutic drugs [38]. In addition, it reduces glucose uptake and lactate production by inhibiting the transcriptional levels of HIF-1α, thereby enhancing mitochondrial oxidative phosphorylation [39]. Curcumin is a compound extracted from the rhizomes of turmeric [40], and its nanosuspension is effective in alleviating the hypoxic and inflammatory states in the TME of triple-negative breast cancer, reducing the stability of HIF-1α and increasing its susceptibility to degradation [41].

The glucose analog 2-DG is phosphorylated by the hexokinase and competes with glucose-6-phosphate to inhibit glycolysis, causing apoptosis due to energy deficiency in cancer cells [42]. Combining 2-DG with radiotherapy or chemotherapy to inhibit glycolysis could enhance the therapeutic effect of radiotherapy and chemotherapy. Therefore, a combination of cell metabolism inhibitors and chemotherapeutic agents can be used to treat breast cancer by targeting HIF-associated adipocyte metabolic pathways.

Regulation of immune metabolism has been known to control cancer progression. CD8^+^ T cells have a very high rate of glycolysis, which is further enhanced by the overexpression of HIF-1α, thus increasing the levels of CD8^+^ T cell immune infiltration and activity. However, increased glycolysis enhances the competition for glucose between adipocytes and cancer cells within the tumor area and immune cells [43], causing the exhaustion or death of immune cells. Studies on programmed cell death protein 1 (PD-1) and T lymphocytes have demonstrated that PD-1 activates T cell apoptosis and suppresses immune responses [44]. The PD-1 blocking therapy improves the competition of T cells for glucose and restores their glycolytic ability, thereby manifesting their immune efficacy [43]. Future therapies could consider combining treatments inhibiting tumor metabolism with agents enhancing the glycolytic ability of T cells to promote optimal anti-tumor immunity.

## Conclusions and future directions

Adipocytes are intricately related to the development of breast tumors, with hypoxia acting as the key crosstalk between adipocytes and their neighboring breast cancer cells. Under hypoxia, metabolic reprogramming of adipocytes allows breast cancer cells to maintain hypoxia.

Cancer cells are highly plastic and their morphology can change dynamically in response to external signals in the microenvironment. In addition, epithelial–mesenchymal transformation increases their resistance to chemotherapy and metastatic capacity through plasticity [45, 46]. Hypoxia, induced either by adipocytes or within the tumor microenvironment, has been identified as a critical factor in promoting cancer stem cell populations and activating pathways associated with increased migration and metastasis. Under hypoxic conditions, HIF-1α regulators increase Snail1 levels and induce EMT by activating the COX-2/PGE2 pathway [47]. Reverse differentiation of cancer cells into epithelial cells is now being used to treat cancer, which greatly reduces the proliferation of cancer cells and increases their sensitivity to chemotherapy [48]. This strategy has led to the development of adipogenesis therapy, which inhibits cancer cell metastasis by transdifferentiating breast cancer cells into adipocytes. Factors affecting breast cancer cell differentiation into adipocytes and intra- and extracellular influences on differentiation are not well understood. Therefore, more research is warranted to discover mechanisms that can be used to selectively induce the differentiation of cancer cells into adipocytes.

The immune impact of HIF-dependent metabolism of adipocytes is highly complex, involving several signaling pathways and multiple cell interaction mechanisms. Therefore, it could be used as a target for cancer immunotherapy. In the future, it is necessary to explore different crosstalk mechanisms of adipocytes and breast cancer cells under hypoxia and strategies related to immunotherapy.
